# A New Complication of Spinal Fusion Surgery for Neuromuscular Scoliosis—Posterior Mediastinal Hematoma: Case Report

**DOI:** 10.1227/neuprac.0000000000000034

**Published:** 2023-04-14

**Authors:** Anthony K. Allam, Alex R. Flores, Darrell S. Hanson, David F. Bauer

**Affiliations:** * Department of Neurosurgery, Baylor College of Medicine, Houston, Texas, USA;; ‡ Houston Methodist Orthopedics and Sports Medicine, Houston, Texas, USA;; § Department of Orthopedic Surgery, Texas Children's Hospital, Houston, Texas, USA;; ‖ Department of Neurosurgery, Texas Children's Hospital, Houston, Texas, USA

**Keywords:** Case report, Hematoma, Posterior mediastinal, Scoliosis correction, Spinal fusion

## Abstract

**BACKGROUND AND IMPORTANCE:**

Neuromuscular scoliosis surgery is affiliated with a high risk of perioperative and postoperative complications. We present the case of a never-before-reported complication: a posterior mediastinal hematoma.

**CLINICAL PRESENTATION:**

We present the case of a 17-year-old female patient with cerebral palsy and neuromuscular scoliosis with a progressive thoracic kyphosis to 85° and levoscoliosis to 13.5° who presented for spinal fusion surgery. Postoperatively, the patient developed persistent tachycardia despite initial resuscitation, anxiolysis, and analgesia. A computed tomography scan was obtained revealing a posterior mediastinal hematoma. The patient was managed with supportive care and recovered well.

**CONCLUSION:**

This case highlights a never-before-reported complication of spinal fusion surgery: a posterior mediastinal hematoma. Although unlikely symptomatic, a posterior mediastinal hematoma should be on the differential diagnosis for postoperative tachycardia. This case entreats us to elucidate the true incidence rate of this complication in the population.

**ABBREVIATION:**
PR: prevalence rate.

Surgical correction of neuromuscular scoliosis is affiliated with a high risk of perioperative and postoperative complications. This is owed to the frequent comorbidities and associated surgical risk factors from the patients' underlying disease pathology.^1^ In a recent meta-analysis, pulmonary complications were found to be the most common category of complication arising from neuromuscular scoliosis correction with a prevalence rate (PR) of 22.71%.^1^ This was followed by implant-related complications (PR: 12.51%), infections (PR: 10.91%), neurological complications (PR: 3.01%), and pseudoarthrosis (PR: 1.88%).^1^ There have been a few cases reports that have noted retroperitoneal and cervical hematomas after spinal fusion surgery; however, there was no reports regarding mediastinal hematomas.^2-10^ Here, we present a previously unreported complication of spinal fusion surgery for neuromuscular scoliosis correction: a posterior mediastinal hematoma. Furthermore, we reviewed the available literature to identify similar cases of mediastinal hematomas following spinal fusion.

This case report has been reported in line with the Surgical Case Report (SCARE) Criteria.^11^

## CLINICAL PRESENTATION

**FIGURE 1.**
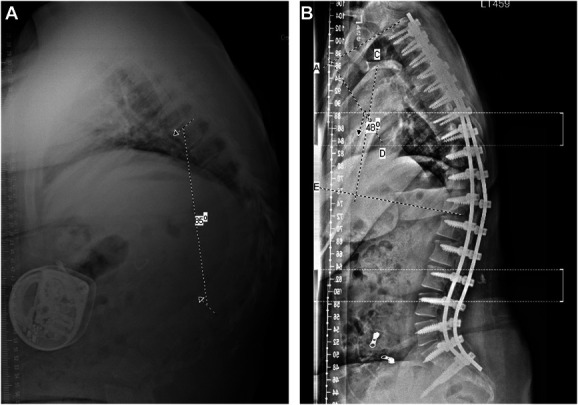
**A**, Preoperative vs **B**, postoperative scoliosis films showing 85° vs 48° of thoracic kyphosis, respectively.

**FIGURE 2.**
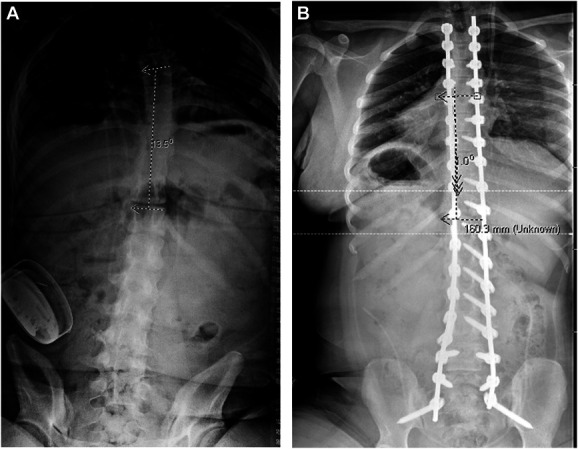
**A**, Preoperative and **B**, postoperative posterior-anterior scoliosis films showing a 13.5° and 1.0° levoscoliosis of the thoracic spine, respectively.

The patient was a 17-year-old female with cerebral palsy and neuromuscular scoliosis with a progressive thoracic kyphosis to 85° and levoscoliosis to 13.5° (Figures 1 and 2). She had a history of obstructive hydrocephalus from prior intraventricular hemorrhage status after ventriculoperitoneal shunt and intrathecal baclofen pump for spasticity. She underwent planned removal of the baclofen pump because of disuse, followed by thoracic (T2) to pelvis segmental fixation and arthrodesis with deformity correction, with ponte osteotomies from T4-L1 by 2 experienced surgeons. Navigation was used for pedicle screw placement. All trajectories were sounded before screw placement, with no cortical breaches noted. Pelvic fixation was placed through an S2-iliac technique. After placement of all screws, a temporary rod was cut and bent with desired kyphosis, and cantilever technique with segmental compression was used to reduce the thoracic kyphosis. Permanent cobalt chromium rods were placed. Intraoperative films showed good correction of the thoracic kyphosis from 85 to approximately 45° with good correction of the mild scoliosis. Posterior spinal fusion was performed with a mixture of auto and allograft. No complications were evident at completion of the surgery.

**FIGURE 3.**
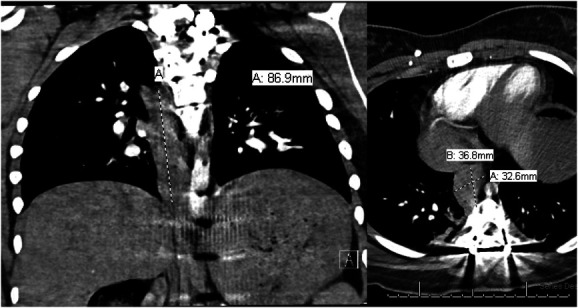
Contrasted computed tomography of the chest, coronal and axial views, showing right eccentric 8.7 cm craniocaudal × 3.3 cm mediolateral × 3.7 cm anteroposterior posterior mediastinal hematoma, extending from T5-T9.

On postoperative day 1 the patient had persistent sinus tachycardia in the 120s despite apparent adequate resuscitation, anxiolysis, and analgesia. Although her respiratory status remained uncompromised, a computed tomography (CT) pulmonary embolism study of the chest was pursued, revealing an 8.7 cm craniocaudal × 3.3 cm mediolateral × 3.7 cm anteroposterior posterior mediastinal hematoma, extending from T5-T9 (Figure 3). There were no pedicle screws abutting the hematoma. There was no active extravasation to indicate a site of bleeding.

Cardiothoracic and vascular surgeries were both consulted, recommending CT angiogram of the abdomen and pelvis to assess the entire aorta. Imaging revealed a decreasing hematoma and no other abnormalities. All pedicle screws were well placed within the bone. The hematoma was managed with supportive care, and the tachycardia ultimately resolved on its own. The hematoma was not felt to be compressive on the heart or the cause of the sinus tachycardia. The patient made an excellent recovery.

The patients/participants provided their written informed consent to participate in this study and for the publication of this case report.

## DISCUSSION

Here, we report a case of a 17-year-old female patient with neuromuscular scoliosis undergoing posterior spinal fusion who developed postoperative tachycardia and found to have a posterior mediastinal hematoma. A systematic review of the literature was performed to identify similar complications secondary to a spinal fusion surgery (**Supplemental Digital Content 1, Literature Review Methods**, http://links.lww.com/NEUOPEN/A58) (**Supplemental Digital Content 2, Preferred Reporting Items for Systematic Reviews and Meta-Analyses (PRISMA) Methods Outline**, http://links.lww.com/NEUOPEN/A59). There have been a few cases of retroperitoneal and cervical hematomas resulting from iatrogenic injury to small blood vessels after lumbar or cervical fusion.^2-10^ However, to the best of our knowledge, there are no other reports of a mediastinal hematoma after spinal fusion.

**FIGURE 4.**
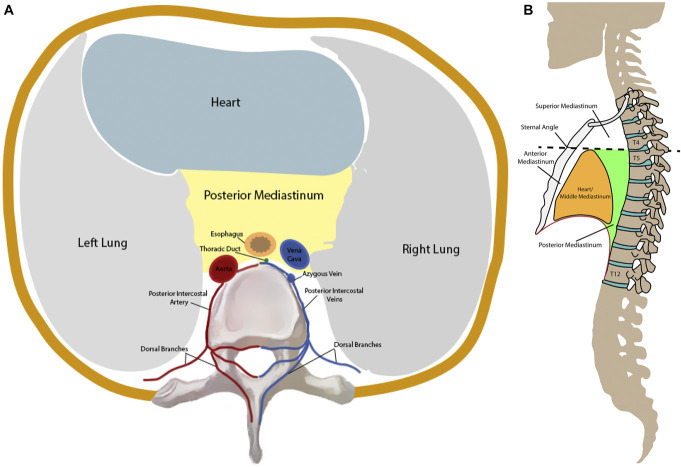
**A**, Axial representation of the thoracic cavity. This portion of the figure is adapted from Bijendra et al^20^ under a Creative Commons Attribution 4.0 International License. **B**, Sagittal representation of the thoracic cavity. *Adapted from an image created by the National Cancer Institute, Public Domain,* https://commons.wikimedia.org/w/index.php?curid=1394201

The posterior mediastinum is a space located posteriorly to the pericardial sac housing important neurovascular structures (eg, descending aorta, vagus nerve, sympathetic trunks, azygous and hemiazygous veins, esophagus, thoracic duct etc.) (Figure 4). The posterior intercostal arteries, branches of the aorta and the superior intercostal artery, provide blood flow to the posterior chest wall in this region. Dorsal branches supply the spine and the muscles of the back. The blood is then drained through the dorsal branches of the posterior intercostal veins into the azygous and hemiazygos veins.

Traditional sources of a posterior mediastinal hematoma include the rupture of the descending aorta, rupture of an aneurysm of the inferior thyroid artery, and vertebral fractures.^12-14^ Certain coagulation abnormalities have been reported to cause posterior mediastinal hematomas as well. However, in our case, we propose that the hematoma was caused by the avulsion of a small tributary/tributaries of the posterior intercostal veins during spinal manipulation for scoliosis correction during surgery. In this case, the hematoma was likely incidental anddiscovered on workup of postoperative tachycardia likely due to combined under-resuscitation, pain/agitation, and mass effect on the heart.^15,16^

In our case, the pathology was managed with a cardiothoracic and vascular consult who preceded with total aorta imaging after the chest CT revealed a posterior mediastinal hematoma. Plain film chest radiography is not recommended because it may not show mediastinal widening clearly.^12,17^ CT, transesophageal echocardiography, or MRI are more sensitive and are indicated if a posterior mediastinal hematoma is suspected.^12,18,19^ The patient was able to recover well with only supportive care. No follow-up imaging was pursued given resolve of tachycardia.

The incidental nature of this suggests that (1) rupture of small azygous/hemiazygos veins during these corrections rarely manifest clinical symptoms; (2) the incidence of rupture and specifically of posterior mediastinal hematomas from these surgeries is likely higher than previously thought.

## CONCLUSION

Although perioperative and postoperative complications are relatively common with neuromuscular scoliosis correction surgery, our case presents a previously unknown complication, a posterior mediastinal hematoma. Although incidental in our case, a posterior mediastinal hematoma should be on the differential diagnosis for postoperative tachycardia. If asymptomatic, as in our case, it can be managed with cardiothoracic and vascular consults, as well as radiographic inspection of the total aorta. Additional research is needed to elucidate the true incidence of this complication.

## Supplementary Material

**Figure:**
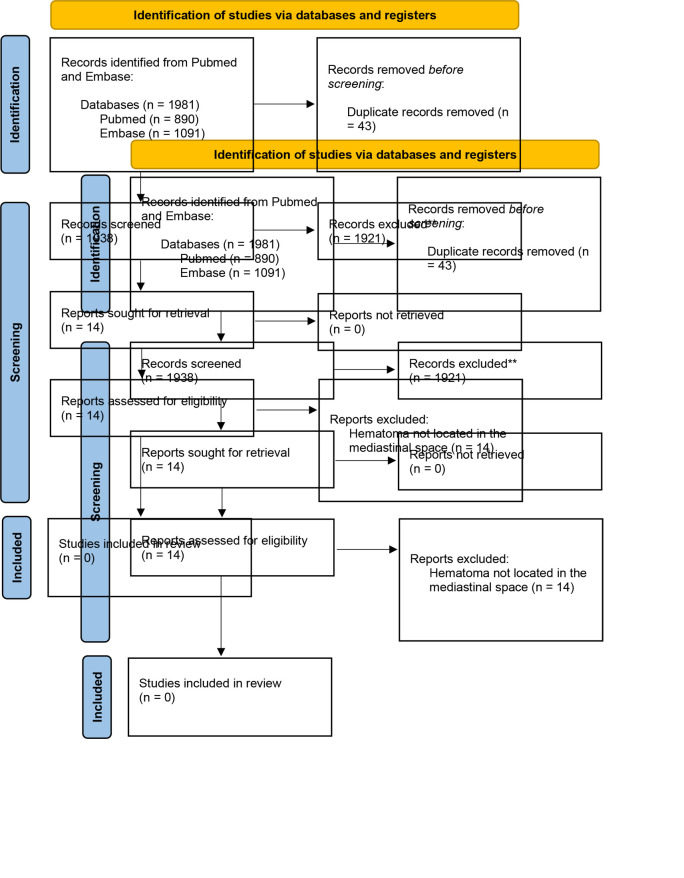


## Supplemental Digital Content

**Supplemental Digital Content 1.** Literature review methods **Supplemental Digital Content 2.** Preferred Reporting Items for Systematic Reviews and Meta-Analyses (PRISMA) methods outline

## COMMENTS

The authors present a complication of a retromediastinal hematoma after correction of a neuromuscular kyphoscoliosis. They suggest it likely is an underreported complication which certainly could be true given the high complication rates associated with neuromuscular scoliosis. Surgical blood loss is typically higher in these cases and fairly common need for blood transfusions/post-op tachycardia may mask the presentation of such a complication. Surgeons should certainly be aware of this unusual complication as a possible outcome after deformity correction. Although the authors suggested a likely etiology of a vessel tear during correction, another possible cause would be a cortical breach during pedicle screw placement which may have injured a vessel but was then corrected intra-op.

**Steven Hwang**

*Boston, Massachusetts, USA*
