# Supplementary material for: A New Complication of Spinal Fusion Surgery for Neuromuscular Scoliosis—Posterior Mediastinal Hematoma: Case Report
Source: Neurosurg Pract. 2023 Apr 14;4(2):e00034. doi: 10.1227/neuprac.0000000000000034 (PMC11810013; doi:10.1227/neuprac.0000000000000034)
Supplement: Supplementary file 1 [file neuopen-4-e00034-s001.docx]

**Supplemental Digital Content 1.** Literature Review Methods. PubMed and Embase were used as the primary databases for electronic article searching. The National Library of Medicine’s PICO (Patient/Population/Problem, Intervention, Comparison, and Outcome) guideline was used to guide the literature search terms. The term “P(hematoma and post operative and mediastinum) I(spinal fusion)” was used, with “C” and “O” being omitted as to maximize the capture of relevant papers and reduce selection bias. The MeSH (Medical Subject Heading) terms used in the search included: hematoma, spinal fusion, post operative, mediastinum, epidural, and subdural. The terms epidural and subdural were used to omit articles not relevant to the search. The formal search consisted of the following structure: “(hematoma AND spinal fusion AND post operative AND mediastinum) NOT (epidural OR subdural)”. Only publications in English were considered. This search yielded a total of 1 result which was then screened through an abstract review to yield a total of 0 papers. We then expanded our search criteria to include any hematomas located extraspinally by eliminating the word “mediastinum”. Our formal search was as follows: “(hematoma AND spinal fusion AND post operative) NOT (epidural OR subdural)”. This search yielded a total of 1938 results which were then screened through an abstract and title review to yield a total of 14 papers. These 14 papers then underwent a full-text review. The final papers were chosen based on the following criteria: 1) the patient must have had a post-operative hematoma, 2) the patient must have undergone spinal surgery, 3) the post-operative hematoma was located between T4-T10, and 4) the study was written in English. Exclusion criteria included the presence of a subdural and/or epidural hematoma. There was a total of 0 papers that fit the inclusion and exclusion criteria present in our search.
